# Disulfidptosis as a key regulator of glioblastoma progression and immune cell impairment

**DOI:** 10.3389/fimmu.2025.1526296

**Published:** 2025-01-30

**Authors:** Yifu Shu, Jing Li

**Affiliations:** Department of Neurosurgery, Taikang Ningbo Hospital, Ningbo, China

**Keywords:** glioblastoma, disulfidptosis, PD-L1, t cell exhaustion, multi-omics

## Abstract

**Background:**

Glioblastoma, associated with poor prognosis and impaired immune function, shows potential interactions between newly identified disulfidptosis mechanisms and T cell exhaustion, yet these remain understudied.

**Methods:**

Key genes were identified using Lasso regression, followed by multivariate analysis to develop a prognostic model. Single-cell pseudotemporal analysis explored disulfidptosis T-cell exhaustion (Tex) signaling in cell differentiation. Immune infiltration was assessed via ssGSEA, while transwell assays and immunofluorescence examined the effects of disulfidptosis-Tex genes on glioma cell behavior and immune response.

**Results:**

Eleven disulfidptosis-Tex genes were found critical for glioblastoma survival outcomes. This gene set underpinned a model predicting patient prognosis. Single-cell analysis showed high disulfidptosis-Tex activity in endothelial cells. Memory T cell populations were linked to these genes. SMC4 inhibition reduced LN299 cell migration and increased chemotherapy sensitivity, decreasing CD4 and CD8 T cell activation.

**Conclusions:**

Disulfidptosis-Tex genes are pivotal in glioblastoma progression and immune interactions, offering new avenues for improving anti-glioblastoma therapies through modulation of T cell exhaustion.

## 1 Introduction

Glioblastoma (GBM), a highly aggressive brain cancer, has a median survival time of 15 months. Current treatments’ limited efficacy, including surgery, radiotherapy, and chemotherapy, underscores the urgent need for innovative therapies and a deeper understanding of GBM’s molecular basis (1–3). Crucially, the tumor microenvironment and mechanisms of immune escape, such as T-cell exhaustion (Tex), play significant roles in glioblastoma (GBM) disease progression and therapy resistance (4, 5). T cell exhaustion is characterized by a progressive loss of effector functions and sustained expression of inhibitory receptors, which impairs the immune system’s ability to effectively combat tumor cells. In gliomas, T cell function diminishes due to persistent antigen exposure and the presence of immunosuppressive factors like TGF-β and IL-10, alongside increased expression of immune checkpoints PD-L1 and CTLA-4 (6–10). These factors collectively result in an impaired immune response and enhanced tumor proliferation (11–13). Recent studies have elucidated the roles of metabolic dysregulation, epigenetic modifications, and chronic antigen exposure in driving T cell exhaustion (14–16). Furthermore, advancements in single-cell technologies have revealed the heterogeneity within exhausted T cell populations, identifying distinct subsets with varying functional states (17, 18). Understanding these complex mechanisms is crucial for developing targeted immunotherapies aimed at reinvigorating exhausted T cells and enhancing anti-tumor immunity (19, 20).

In addition, recent studies have begun to unravel the complex genetic and epigenetic landscape of cancer (21–24), yet the role of emerging cellular processes, such as disulfidptosis—a novel cell death pathway—and the intricate dynamics of the tumor immune microenvironment remain largely underexplored (25). As a newly characterized type of regulated cell death (RCD), disulfidptosis is considered to be closely related to the occurrence and development of tumors as ferroptosis and cuproptosis death, which were fully explored in the past, which is incited by the aberrant intracellular buildup of disulfides (26), and this procedure cannot be mitigated by previous inhibitors of cell death (27). Its linkage between cellular metabolism and fate and its significant impact on tumor immune responses is arousing great interest (28, 29). It is found that under a glucose starvation situation, the expression of solute carrier family 7 member 11 can induce the abnormal accumulation of cystine and other disulfides (30, 31). The formation of these disulfide bonds between actin cytoskeletal results in the collapse of the cytoskeleton structure and, eventually, cell death. Further, the treatment of glucose transporter (GLUT) inhibitors can trigger disulfidptosis, which indicates that the inducement of disulfidptosis might be a promising therapeutic strategy (26). It is also reported that the disulfidptosis procedure not only establishes a linkage between cellular metabolism and cellular destiny but also demonstrates a conspicuous association with the immune response within the tumor microenvironment (32, 33). Emerging research has shown that many cancer cells experience oxidative stress, leading to disulfide metabolism disorders that affect cancer cell survival and proliferation (34–37). Additionally, disulfide metabolism in cancer cells is also associated with biological behaviors such as drug resistance, metastasis, and immune escape (38–40). Understanding the interplay between disulfidptosis and GBM may provide insights into the complex biology of GBM and help identify potential therapeutic targets, ultimately improving the outcomes of GBM patients.

Building upon these insights, our study investigates the intricate interplay between disulfidptosis and Tex within the glioblastoma microenvironment, aiming to uncover novel therapeutic targets and enhance treatment outcomes for GBM patients. By leveraging the relationship between disulfidptosis and Tex, we have developed a robust prognostic model for GBM survival and identified key targets that could potentiate T-cell-mediated tumor control. This integrative approach not only facilitates the prediction of patient outcomes but also paves the way for precision therapies tailored to individual molecular profiles. Ultimately, our research seeks to advance immunotherapeutic strategies in combating glioblastoma, offering promising avenues for personalized medicine and improved clinical efficacy.

## 2 Materials and methods

### 2.1 Immunofluorescence assay

For LN229 cell replication, adhere the cells to glass coverslips until they reach 30% confluency, then fix them with 4% formaldehyde in phosphate-buffered saline (PBS) for 10 minutes at room temperature. After washing with PBS, increase membrane permeability by treating with 0.1-0.5% Triton X-100 in PBS for 5 minutes. Block nonspecific binding by incubating with 5% bovine serum albumin (BSA) or serum for 30 minutes. Next, incubate with the primary antibody, diluted in blocking solution, for 1 hour at room temperature or overnight at 4°C. After washing with PBS, a fluorescent secondary antibody was applied for 1 hour in the dark, followed by a final PBS wash (41–43). Mount the coverslip and examine the cellular signals using a fluorescence microscope (44, 45).

### 2.2 Apoptosis detection using flow cytometry

Apoptosis was assessed using the Annexin V-FITC kit from BD Biosciences, USA. Cells were incubated with Annexin V-FITC for 15 minutes, followed by a 5-minute incubation with propidium iodide (PI), both in the dark. Flow cytometric analysis was performed with BD Biosciences equipment, and data were analyzed using FlowJo software.

### 2.3 Cell invasion and migration assays

Cell invasion was evaluated using Matrigel-coated Transwell inserts. LN229 cells (5 x 10^5 cells/ml) were seeded in the upper compartment and incubated for 36 hours at 37°C in 5% CO_2_. After incubation, cells adhering to the upper membrane were fixed with 4% formaldehyde, stained with crystal violet, washed with PBS, and examined microscopically. The invasion was quantified by counting cells that migrated through the membrane in five random fields (46). A wound-healing assay was conducted to study the effect of disulfidptosis-Tex on cell migration. A scratch was made in the monolayer at 0 hours, detached cells were removed with PBS, and images were taken after 36 hours for analysis (47).

### 2.4 Evaluation of mitochondrial membrane potential

The mitochondrial membrane potential (Ψm) was evaluated using the ‘Ψm Assay JC-1 Kit’ (Solarbio, M8650, China), employing JC-1 as a fluorescent probe. When the membrane potential is high, JC-1 accumulates within the mitochondrial matrix, leading to the emission of red fluorescence. Conversely, at reduced potentials, JC-1 forms monomers that emit green fluorescence.

### 2.5 Measurement of reactive oxygen species detection

The Reactive Oxygen Species (ROS) levels were measured using the Reactive Oxygen Species Assay Kit (Solarbio, CA1410, China) with DCFH-DA as the fluorescent probe. ROS converts the non-fluorescent DCFH to fluorescent DCF, which is then analyzed to determine intracellular ROS concentrations.

### 2.6 Transcriptomic and clinical data analysis for glioblastoma

Transcriptomic and comprehensive clinical data for the TCGA-GBM cohort were sourced from the GDC portal (https://portal.gdc.cancer.gov/). The study focused on entries that provided both extensive clinical records and transcriptomic data (48, 49). Additionally, the CGGA database was used for whole-genome expression profiles with corresponding clinical information for GBM (50).

### 2.7 Single-cell transcriptomic analysis

Using Seurat package version 4.2.0, the pre-filtered single-cell dataset was imported (51, 52). Data normalization was performed using the ‘NormalizeData’ function. Post-normalization, genes with significant variation were identified by balancing average expression levels and dispersion metrics. The ‘FindClusters’ function, a graph-based clustering tool using a modularity optimization algorithm from shared nearest neighbors, delineated 19 distinct clusters from 33 principal components at a resolution of 0.2. Differentially expressed genes (DEGs) in each cluster were determined using ‘FindAllMarkers’ with default settings in Seurat.

### 2.8 Cell communication profiling assessment

Cell Communication Profiling via single-cell analysis ligand-receptor interactions among various cell types were analyzed to identify unique signaling pathways (53). The ‘CellChat’ tool quantified and estimated the probability of intercellular signaling interactions, applying default parameters with a significance threshold of P ≤ 0.05 and adjustments for multiple testing using the Benjamini-Hochberg procedure (54). We also assessed the expression of ten disulfidptosis-Tex-related genes across different glioblastoma cell types using the AUCell scoring method (55, 56). Twelve cell types identified in the scRNA data were analyzed, categorizing cells with AUCell scores above 0.3 as high disulfidptosis-Tex activity and those with lower scores as reduced activity.

### 2.9 Enrichment analysis

Disulfidptosis-Tex’s role in glioblastoma was assessed using ‘ssGSEA’ to calculate gene enrichment scores in individual samples (57–59). Using ‘surv_cutpoint’, samples were categorized into low or high disulfidptosis-Tex enrichment groups. Intersection analysis identified cell types with significant disulfidptosis-Tex activity and contrasted gene enrichment between the groups. T cell exhaustion-linked DEGs in GBM were identified. Gene Ontology (GO) and Kyoto Encyclopedia of Genes and Genomes (KEGG) analyses with ‘clusterProfiler’ revealed pathways enriched among these DEGs (60–62).

### 2.10 Prognostic evaluation in glioblastoma

The prognostic relevance of disulfidptosis-Tex-associated DEGs in glioblastoma was evaluated using univariate Cox regression to analyze their correlation with patient overall survival (OS) (63–65). Genes with a P-value < 0.05 were selected for further analysis (66). The study used 268 tumor samples, split into training and validation cohorts in a 7:3 ratio, with 106 samples in the latter. A prognostic model was developed using the LASSO Cox regression method via the ‘glmnet’ R package (67, 68), refining the list of potential genes.

### 2.11 Differential expression and functional analysis

Differential expression between high- and low-risk glioblastoma groups was analyzed using the ‘limma’ R package (69, 70). Gene Set Enrichment Analysis (GSEA) of log2 FC-ranked genes was performed with ‘clusterProfiler’ (71–73), and functional differences were examined using ‘GSVA’ (74, 75). Results were visualized with ‘pheatmap’.

### 2.12 Immune pathway activity and immune cell infiltration analysis

ssGSEA analyses were performed using the ‘gsva’ package in R to evaluate immune pathway activities in the study’s samples, utilizing established molecular markers (76). As an enhancement of GSEA, ssGSEA calculates enrichment scores for individual gene set pairs across different samples (77). These scores reflect the coordinated regulation of genes, either upregulated or downregulated, within specific gene sets for each sample. Gene expression data from all GBM samples were used to compute enrichment scores for 28 distinct immune cell types, derived from the TISIDB database (http://cis.hku.hk/TISIDB/index.php) (78). Variations in immune cell infiltration levels between low- and high-risk groups were visualized using the ‘ggplot2’ R package (79, 80).

### 2.13 Statistical analyses

Statistical analyses were performed using R software (version 4.1.3) and GraphPad Prism 8.0. A P-value of <0.05 was considered statistically significant (81). In the graphs, the symbols *, **, and *** represent P-values of <0.05, <0.01, and <0.001, respectively.

## 3 Results

### 3.1 Single-cell RNA sequencing reveals disulfidptosis-Tex-associated gene expression in glioblastoma and identifies immune cell subtypes

**Figure 1:**
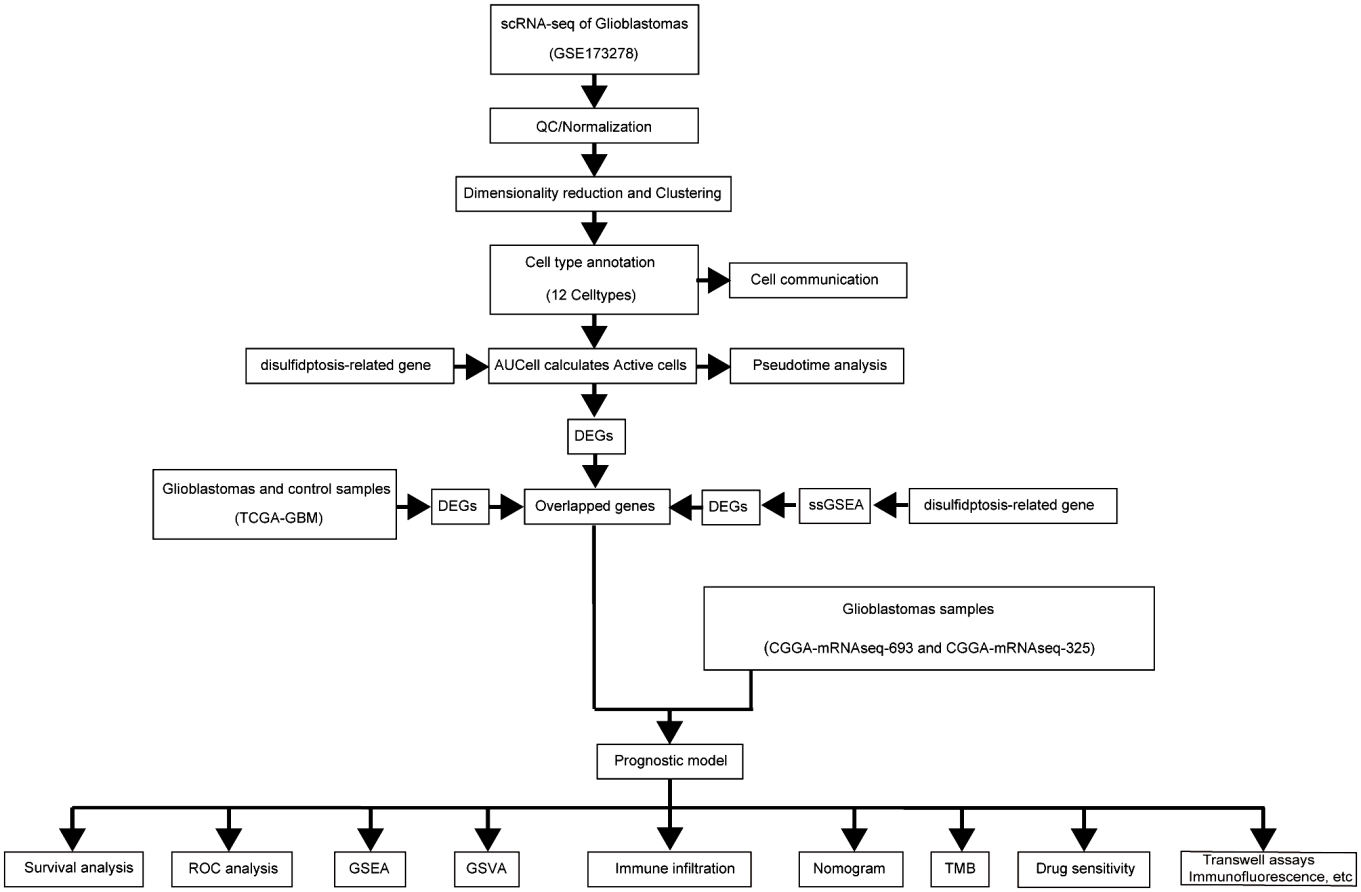
Workflow diagram of study.

**Figure 2:**
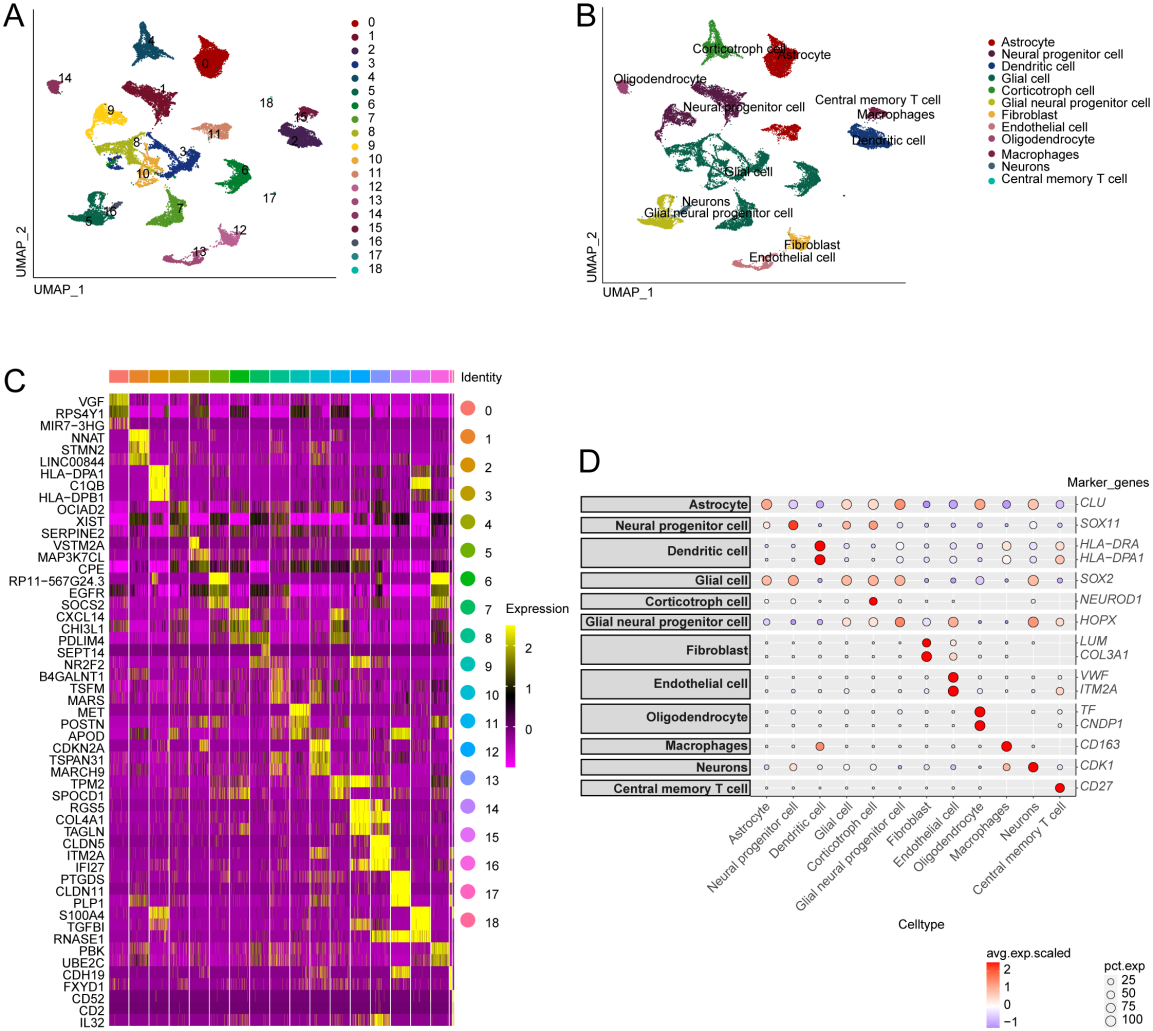
Single-cell analysis of genes associated with disulfidptosis-Tex in glioblastoma. **(A)** Distribution of glioblastoma cell subpopulations. **(B)** Annotation of the different glioblastoma cell subpopulations. **(C)** Gene expression profiles are specific to each cluster. **(D)** Expression levels of disulfidptosis-Tex genes across various cell types.

Single-cell RNA sequencing has significantly enhanced our understanding of the cellular composition of glioblastoma. In this study, we utilized the single-cell RNA sequencing dataset GSE173278 to explore the genes exhibiting elevated expression linked to disulfidptosis-Tex (**Figure 1**). The analysis of single-cell transcriptomes revealed 19 distinct clusters across 29,543 cells, as shown in the UMAP plot (**Figure 2A**). Cell surface markers were also used to identify 12 unique cell subtypes, including dendritic cells, central memory T cells, and macrophages (**Figures 2B, D**). Further investigation focused on disulfidptosis-Tex-associated differential expression genes (DEGs) expression patterns within these subtypes (**Figure 2C**).

### 3.2 Endothelial cell enrichment of disulfidptosis-Tex in glioblastoma

**Figure 3:**
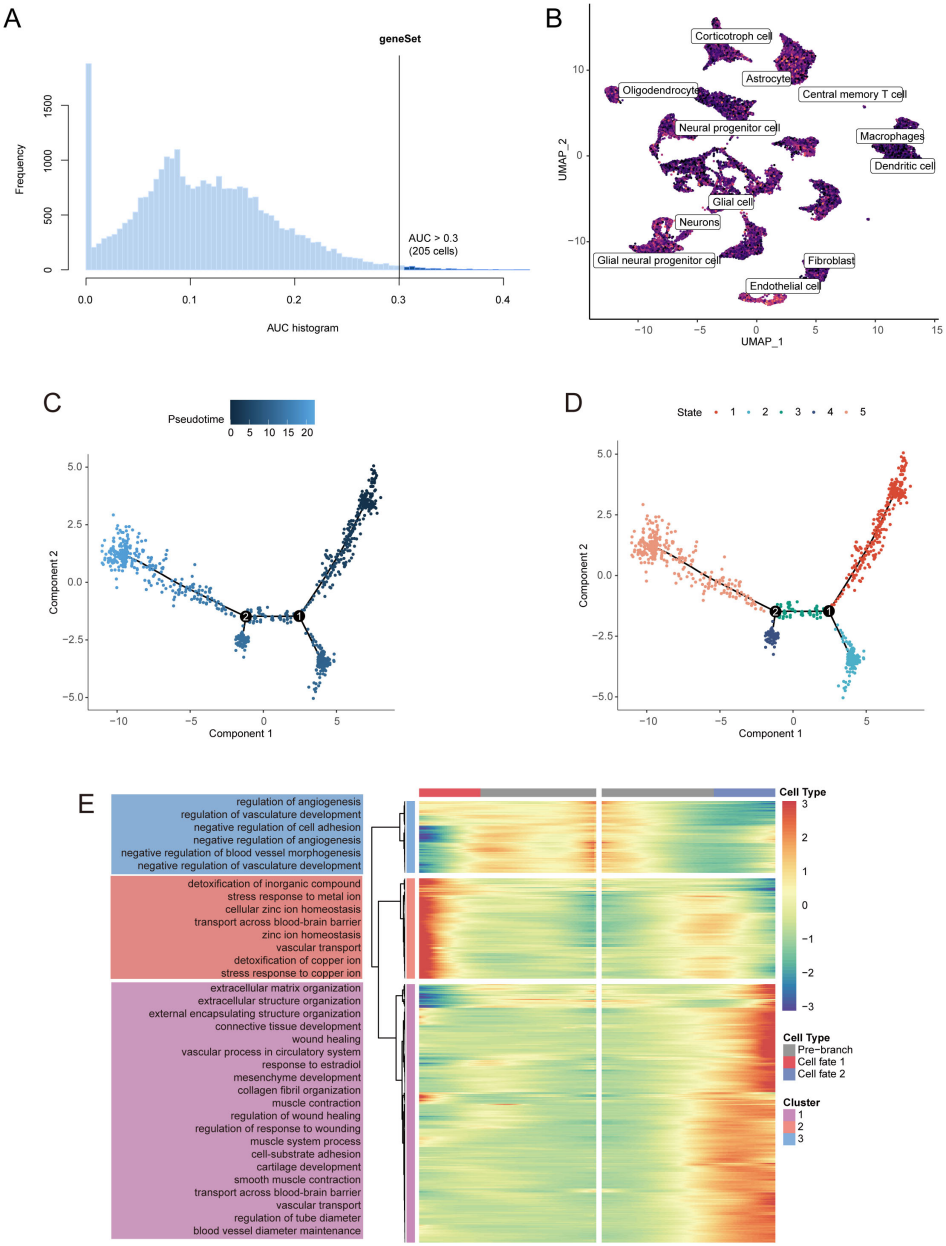
Pseudotime analysis identifies key disulfidptosis-Tex genes implicated in glioblastoma progression. **(A)** AUC scores for disulfidptosis-Tex activity. **(B)** UMAP-based chromatic map displaying the activity scores of disulfidptosis-Tex. **(C)** Pseudotime trajectory analysis. **(D)** Pseudotime trajectories segmented using Monocle2. **(E)** Expression patterns of differentially expressed genes (DEGs) across distinct cell branches.

**Figure 3A** illustrates the identification of 205 cells exhibiting active disulfidptosis-Tex. The UMAP representation of these cells revealed a significant prevalence of endothelial cells (ECs) (**Figure 3B**). Notably, ECs showed the strongest association with disulfidptosis-Tex, indicating a marked accumulation of this marker within tumor-associated ECs.

### 3.3 Pseudotime analysis reveals core disulfidptosis-Tex gene driving glioblastoma progression

Pseudotime analysis of endothelial cells (ECs) in glioblastoma revealed the key role of disulfidptosis-Tex genes in tumor progression (**Figure 3C**). Five distinct transcriptional states were identified along the trajectory (**Figure 3D**). Further analysis of these genes showed their involvement in ‘angiogenesis regulation’ and ‘extracellular matrix organization’ (**Figure 3E**).

### 3.4 Key signaling pathways in cell communication interaction

**Figure 4:**
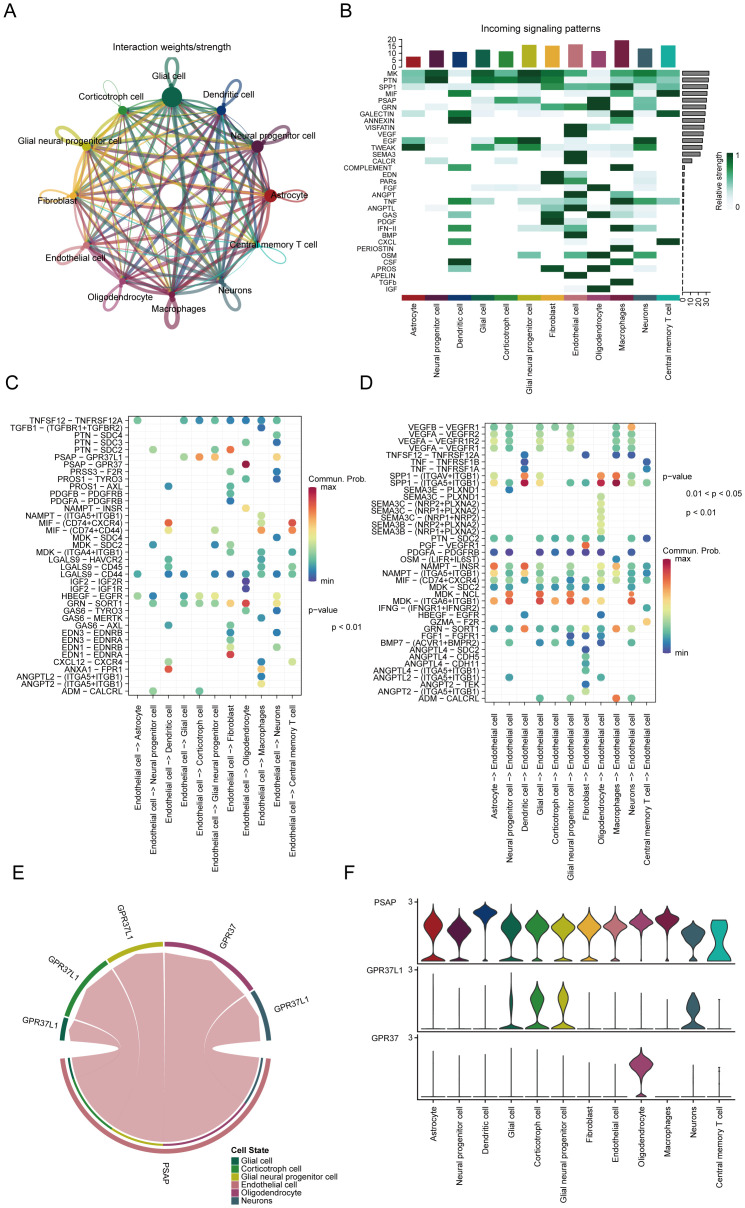
Key signaling pathways involved in cell communication interactions. **(A)** Profiling of cell communication through single-cell analysis. **(B)** Predicted incoming signaling pathways. **(C)** Potential outgoing signaling pairs. **(D)** Predicted incoming signaling pairs. **(E)** Signaling pair interactions. **(F)** Distribution of receptor expression.

To investigate the roles of various cell populations in glioblastoma, we conducted an analysis of intercellular communication, which revealed significant interactions between glioblastoma cells and immune cells, including central memory T cells and macrophages (**Figure 4A**). Both outgoing and incoming signals were examined, alongside relevant ligand-receptor pairs across 12 distinct cell types. Key signaling pathways identified in this analysis included SPP1, PTN, MK, PSAP, GRN, and MIF (**Figure 4B**). Further analysis of the signaling pairs highlighted the PSAP-GPR37 pathway as the predominant interaction in endothelial cells, facilitating communication with oligodendrocytes and dendritic cells (**Figure 4C**). Signaling pathways from oligodendrocytes and macrophages to endothelial cells were also identified (**Figure 4D**). PSAP was found to be expressed across all 12 cell types (**Figure 4E**), while GPR37L1 was predominantly present in corticotroph cells, and GPR37 showed the highest expression in oligodendrocytes (**Figure 4F**).

### 3.5 Identification of differentially expressed genes in disulfidptosis-Tex active subgroups

**Figure 5:**
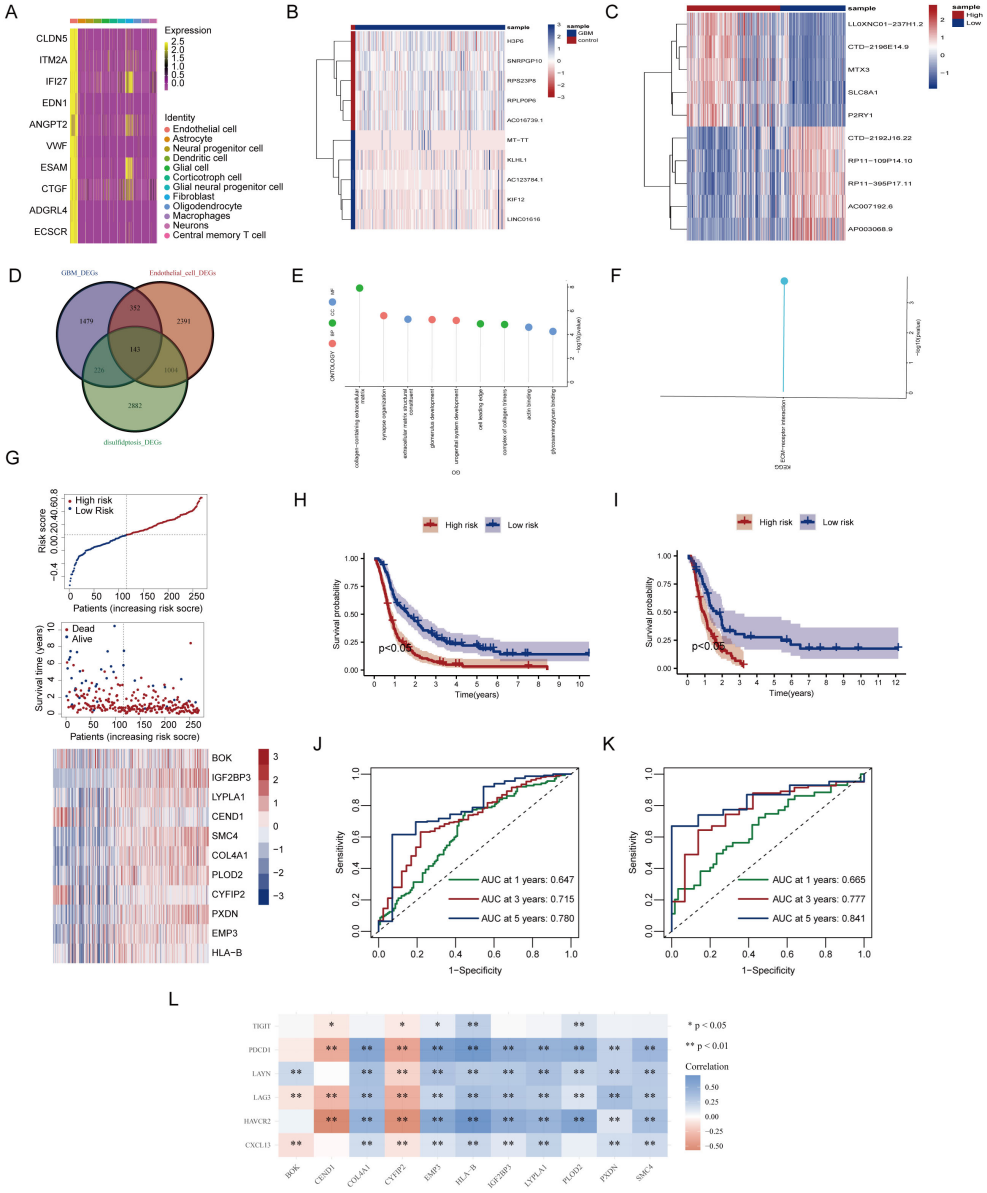
Development of a prognostic signature based on disulfidptosis-Tex. **(A)** Differentially expressed genes (DEGs) in glioblastoma. **(B)** The top 10 genes with the most significant differential expression between glioblastoma and normal control samples. **(C)** Top 10 genes differentially expressed between glioblastoma subgroups with high and low disulfidptosis gene enrichment. **(D)** Venn diagram illustrating gene overlap. **(E)** Gene Ontology (GO) enrichment analysis. **(F)** Kyoto Encyclopedia of Genes and Genomes (KEGG) pathway enrichment analysis. **(G)** LASSO analysis. **(H)** Kaplan-Meier **(K-M)** survival curves for high-risk and low-risk glioblastoma patients in the training cohort. **(I)** Kaplan-Meier **(K-M)** survival curves for high-risk and low-risk glioblastoma patients in the validation cohort. **(J)** Time-dependent Receiver Operating Characteristic (ROC) curves in the training cohort model. **(K)** Time-dependent Receiver Operating Characteristic (ROC) curves in the validation cohort model. **(L)** Correlation analysis between disulfidptosis and exhausted T cells.

In glioblastoma, endothelial cells with the highest disulfidptosis-Tex activity were characterized by 3,890 differentially expressed genes (DEGs) compared to other cell types (**Figure 5A**). A comparison between glioblastoma tissues and normal controls revealed 2,200 DEGs, with a heatmap showing the top five upregulated and downregulated genes (**Figure 5B**). Stratification based on disulfidptosis-Tex activity identified an additional 4,255 DEGs, with the top five genes in the high-activity group also highlighted (**Figure 5C**). An intersection analysis of these groups identified 143 key genes (**Figure 5D**). Enrichment analysis focused on Gene Ontology (GO) and KEGG pathways. GO analysis showed significant enrichment in processes such as synapse organization and urogenital system development, along with molecular functions like glycosaminoglycan binding and ECM structural components (**Figure 5E**). KEGG pathway analysis revealed notable enrichment in ECM-receptor interactions (**Figure 5F**).

### 3.6 Development of the disulfidptosis-Tex-based prognostic signature

A LASSO-Cox regression analysis was performed to assess the impact of disulfidptosis-Tex genes on glioblastoma survival, resulting in a model consisting of 11 key genes (**Figure 5G**). This model successfully stratified patients into high- and low-risk groups, with the high-risk group demonstrating higher mortality rates and upregulated prognostic genes (**Figure 5H**). Kaplan-Meier survival curves further confirmed that the high-risk group had a worse prognosis compared to the low-risk group (**Figure 5I**). The model’s ability to predict patient outcomes was evaluated using ROC curves, achieving AUC values of 0.780 for 5-year survival in the training set (**Figure 5J**) and 0.841 in the validation set (**Figure 5K**). Notably, SMC4 showed a strong negative correlation with T cell exhaustion genes (**Figure 5L**).

### 3.7 Disulfidptosis-Tex affects migration ability of glioblastoma cell

**Figure 6:**
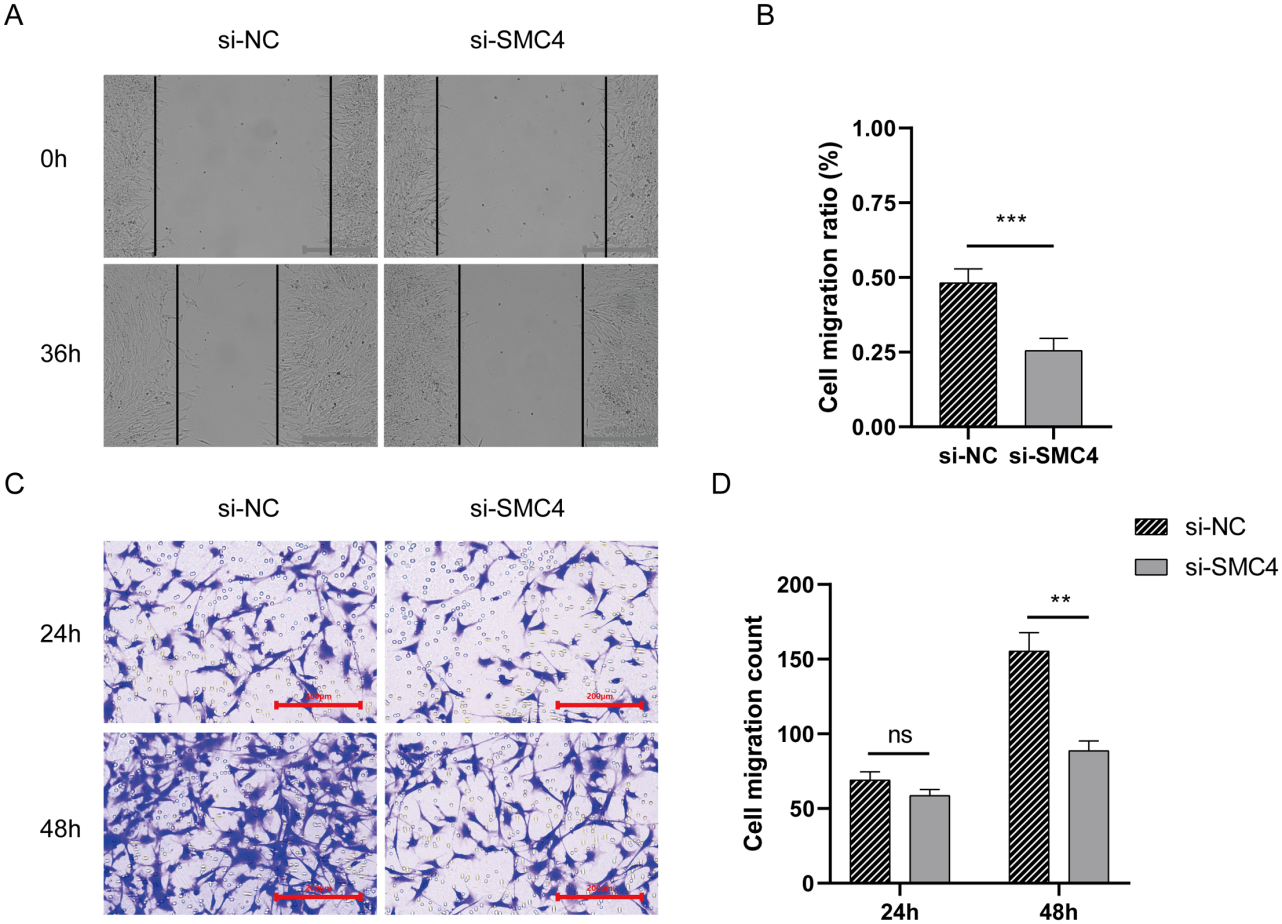
The impact of disulfidptosis-Tex on the migratory capacity of glioblastoma cells. **(A, B)** Wound-healing assay. **(C, D)** Transwell migration assay. ***p < 0.001, **p < 0.01.

We investigated the role of SMC4, a key gene in the disulfidptosis-Tex-related prognostic model, in the migratory behavior of glioblastoma cells. Wound-healing assays showed that silencing SMC4 reduced cell migration at 36 hours compared to controls (**Figures 6A, B**). While transwell assays at 24 hours showed no significant differences between the SMC4-silenced and control groups (**Figure 6C**), at 48 hours, the migration of SMC4-knockdown cells was significantly lower than in the control group (**Figure 6D**).

### 3.8 Enrichment analysis unveiled intricate network influenced by disulfidptosis-Tex in glioblastoma

**Figure 7:**
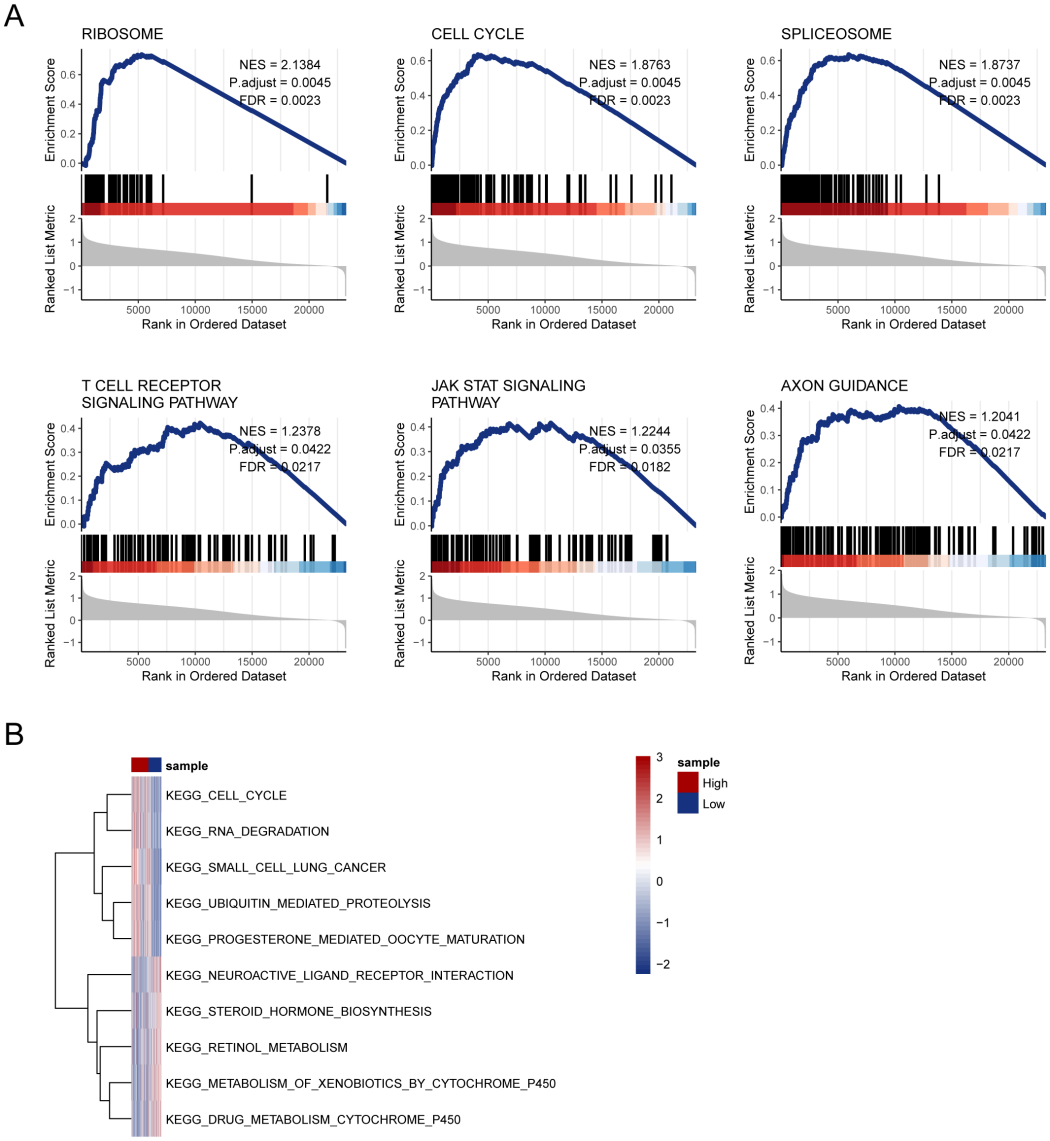
Enrichment analysis unveiled an intricate network influenced by disulfidptosis-Tex in glioblastoma. **(A)** GSEA analysis. **(B)** Heatmap of pathway enrichment differences between high- and low-risk groups from GSVA analysis.

Gene Set Enrichment Analysis (GSEA) was used to explore the underlying mechanisms of the 11-gene disulfidptosis-Tex model. The results revealed significant enrichment in six key pathways, including the T cell receptor signaling pathway, in the high-risk subgroup (**Figure 7A**). Additionally, GSVA was performed using the same MsigDB pathway data, identifying five pathways with the most pronounced differences between high- and low-risk subgroups. A heatmap of these variations revealed a notable enrichment of drug metabolism processes in glioblastoma patients with low disulfidptosis-Tex activity (**Figure 7B**). These findings suggest that disrupting disulfidptosis-Tex could influence drug metabolism and sensitivity in glioblastoma cells.

### 3.9 Connection between disulfidptosis-Tex and immune infiltration

**Figure 8:**
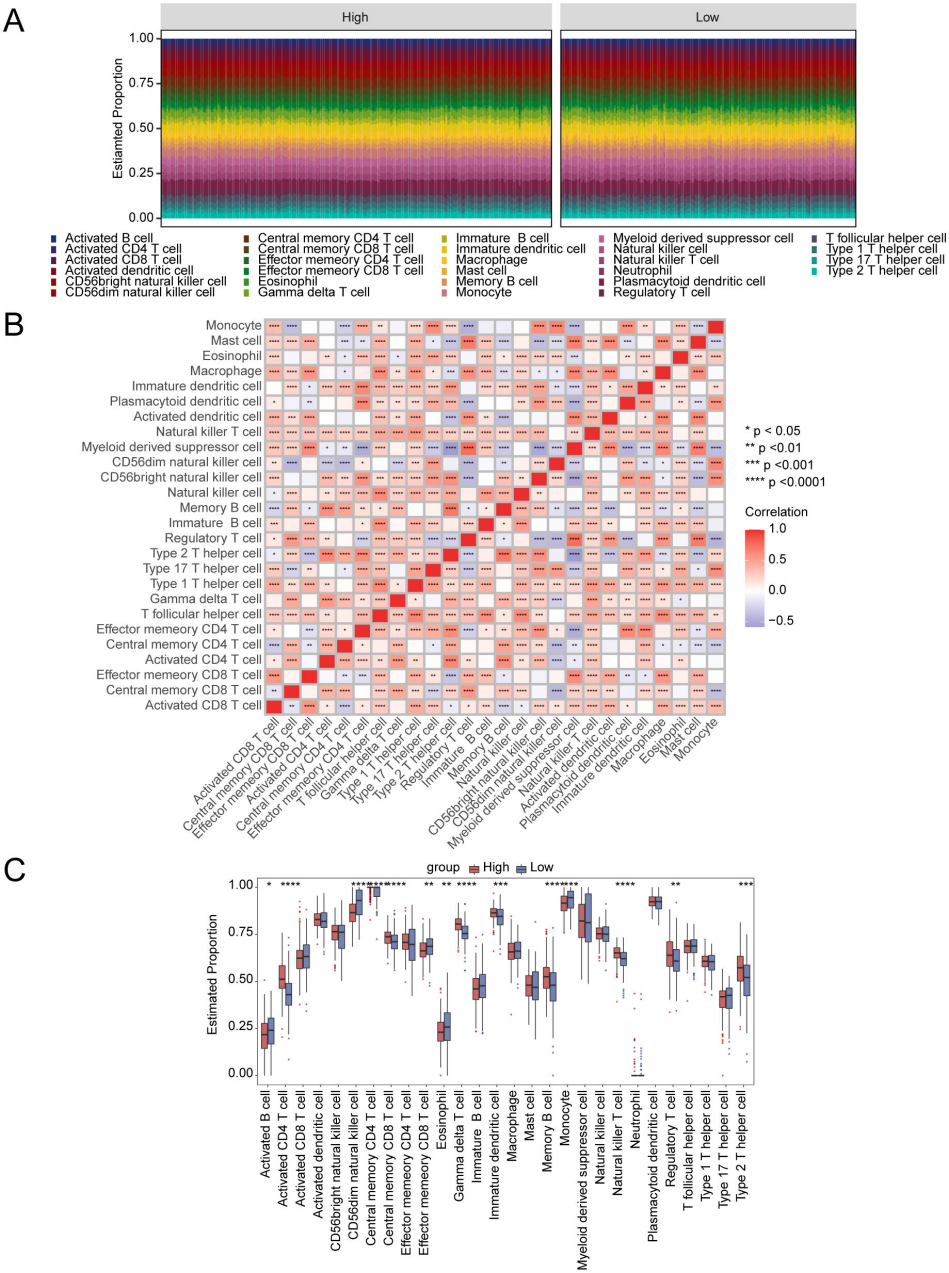
Connection between disulfidptosis-Tex and immune infiltration. **(A)** The relative proportions of immune cells across all glioblastoma samples. **(B)** Correlation matrix of immune cells. **(C)** The proportions of immune cells between high- and low-risk groups. ****p < 0.0001, ***p < 0.001, **p < 0.01, *p < 0.05.

**Figure 9:**
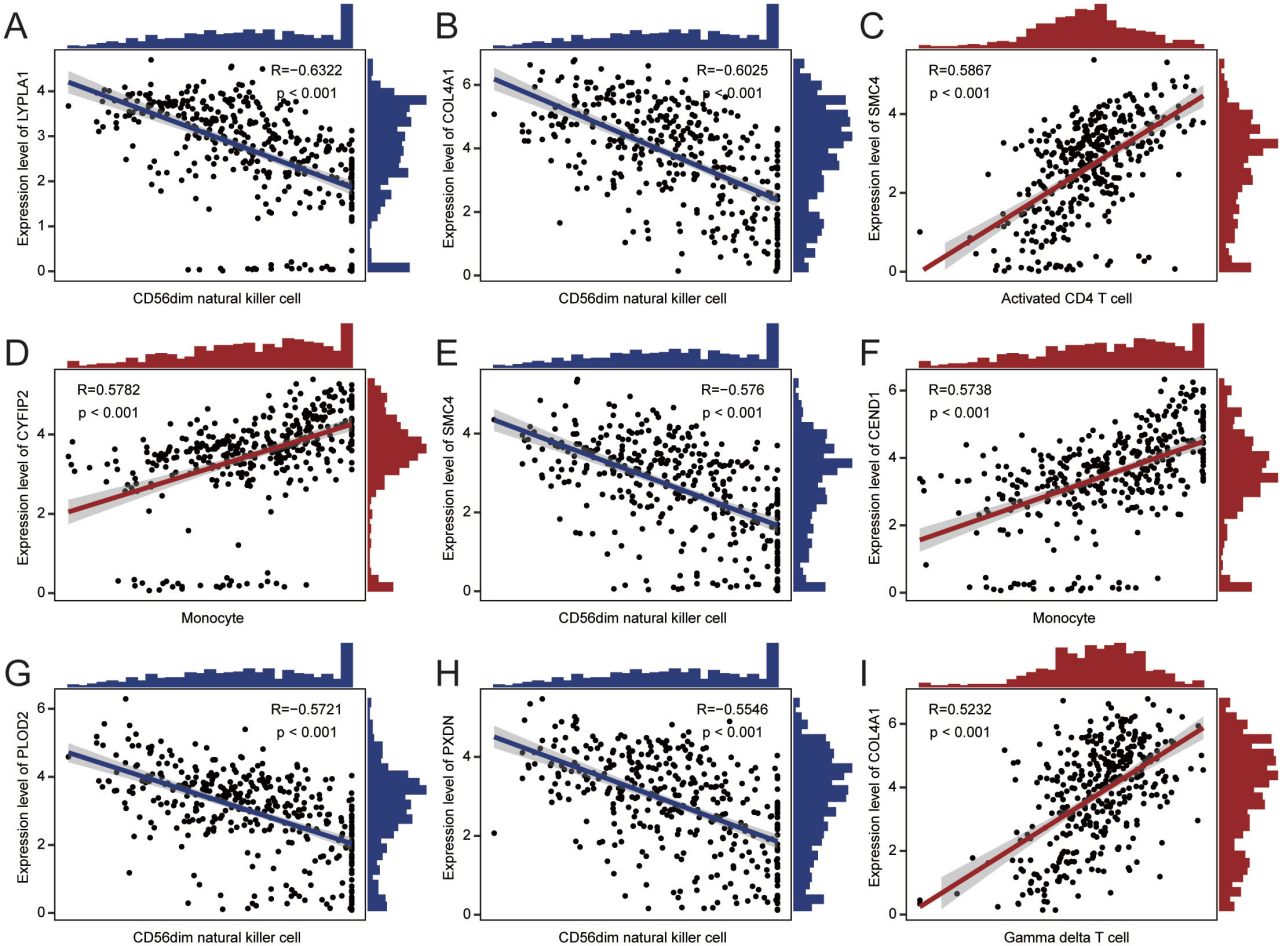
Correlation scatter plots. The correlations between prognostic genes and specific immune cell types. **(A)** LYPLA1 and CD56dim natural killer cells. **(B)** COL4A1 and CD56dim natural killer cells. **(C)** SMC4 and activated CD4 T cells. **(D)** CYFIP2 and monocytes. **(E)** SMC4 and CD56dim natural killer cells. **(F)** CEND1 and monocytes. **(G)** PLOD2 and CD56dim natural killer cells. **(H)** PXDN and CD56dim natural killer cells. **(I)** COL4A1 and gamma delta T cells.

The relationship between immune cell infiltration and tumor progression was demonstrated by analyzing 28 immune cell types in both high- and low-risk subgroups, revealing a link with disulfidptosis-Tex (**Figure 8A**). Notably, activated and central memory CD8 T cells showed a significant negative correlation (**Figure 8B**). Immune cell infiltration differed markedly between risk groups, especially in regulatory T cells (**Figure 8C**). Additionally, strong correlations were found between prognostic genes and specific immune cells (**Figures 9A-I**). SMC4 was positively correlated with activated CD4 T cells (**Figure 9D**) and negatively correlated with CD56dim NK cells (**Figure 9E**). These findings suggest that disulfidptosis-Tex influences the infiltration of CD56dim NK cells and various T cell subsets, emphasizing the role of these genes in modulating tumor microenvironment interactions.

### 3.10 Drug sensitivity prediction and validation

**Figure 10:**
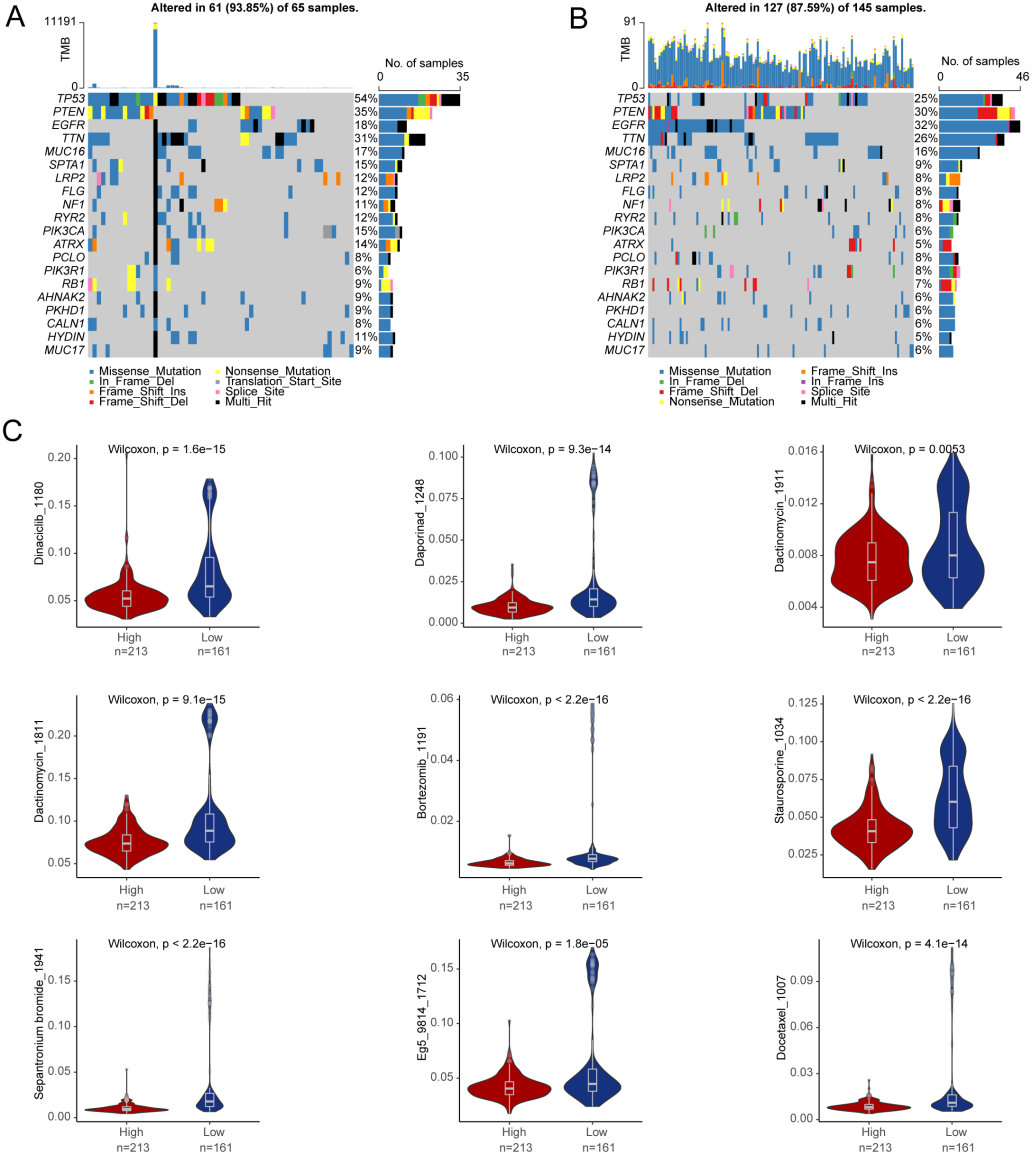
Drug sensitivity prediction. **(A, B)** The top 20 genes with the highest mutation frequency were in the high-risk group and in the low-risk group. **(C)** Differences in drug sensitivity between high- and low-risk groups.

To better understand the prognostic value of the risk score signature in predicting patient outcomes, we examined mutations in glioblastoma-specific genes, focusing on the 20 most frequently mutated genes. TP53 mutations were the most common across both subgroups, followed by PTEN mutations (**Figures 10A, B**). We also evaluated whether risk scores could predict chemotherapeutic responses in glioblastoma patients. Clinical trials were performed testing drugs such as Dinaciclib, Bortezomib, and Docetaxel (**Figure 10C**). The results indicated that patients with higher risk scores exhibited increased sensitivity to these drugs, suggesting that they may be promising treatment options for high-risk glioblastoma patients.

### 3.11 Inhibiting disulfidptosis-Tex induces glioma cell sensitivity to drugs and increases PD-L1 level

**Figure 11:**
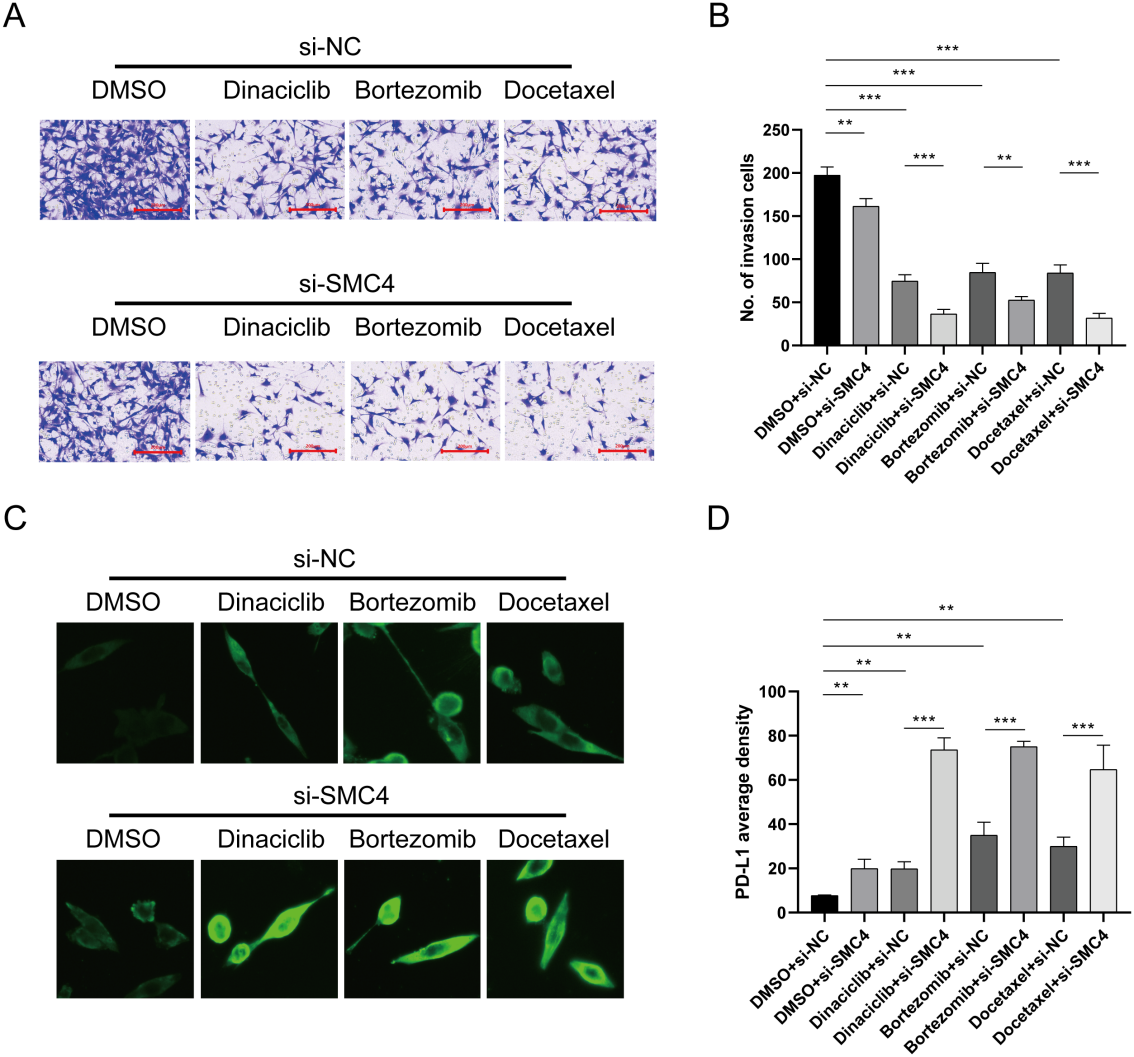
SMC4 regulates PD-L1 expression level. **(A)** Transwell assay of invasion ability. **(B)** Results of invasion cell amounts. **(C)** Cellular immunofluorescence experiment. **(D)** SMC4, in combination with the drug, promotes the upregulation of PD-1 in cells. ***p < 0.001, **p < 0.01.

We also evaluated drug sensitivity using Dactinomycin, Bortezomib, and Docetaxel. The results showed that combining si-SMC4 with these drugs significantly reduced the invasiveness of LN299 cell lines compared to single-agent treatments (**Figures 11A, B**), highlighting the role of disulfidptosis-Tex in glioma metastasis. Interestingly, all the drugs tested were found to upregulate PD-L1 expression in LN299 cells (**Figure 11C**). Further investigation revealed a significant increase in PD-L1 expression when LN299 cells were treated with the si-SMC4-drug combination (**Figure 11D**).

### 3.12 Inhibiting disulfidptosis-Tex induces mitochondrial membrane potential and ROS production in LN299 cells

**Figure 12:**
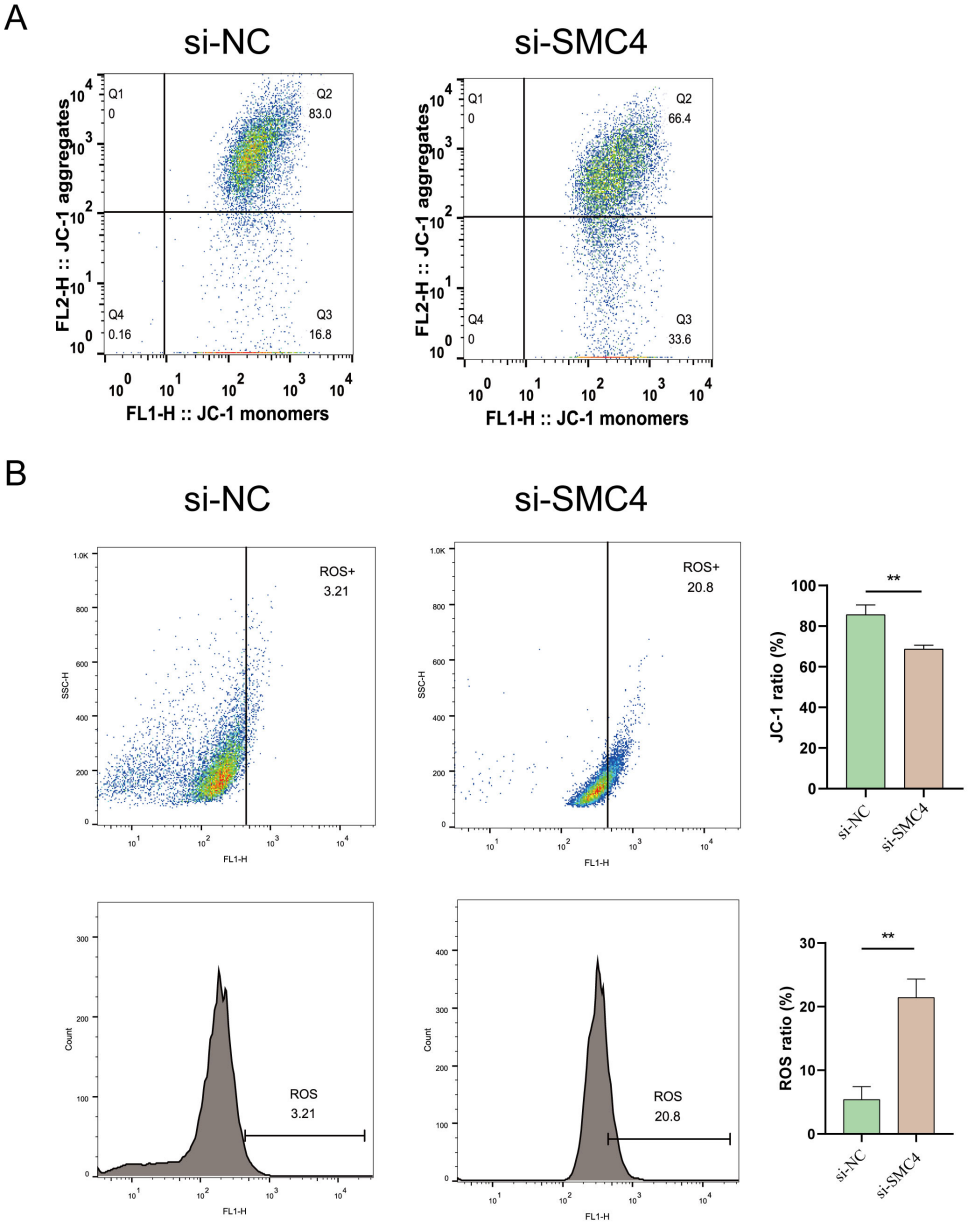
Impact of SMC4 on Mitochondrial Dysfunction and Membrane Potential in Glioblastoma Cells. **(A)** Detection of Mitochondrial membrane potential **(B)** Assessment of ROS production. **p < 0.01.

To evaluate mitochondrial dynamics, LN299 cells were stained with JC-1 and divided into two groups: control and SMC4-interfered. Flow cytometry analysis revealed a significant reduction in mitochondrial membrane potential in the SMC4-interfered cells compared to controls (**Figure 12A**). Additionally, there was a marked increase in reactive oxygen species (ROS) accumulation in these cells (**Figure 12B**). These results suggest that SMC4 plays a regulatory role in maintaining mitochondrial integrity.

### 3.13 SMC4 inhibition reduces the ability of LN299 cells to activate T cells

**Figure 13:**
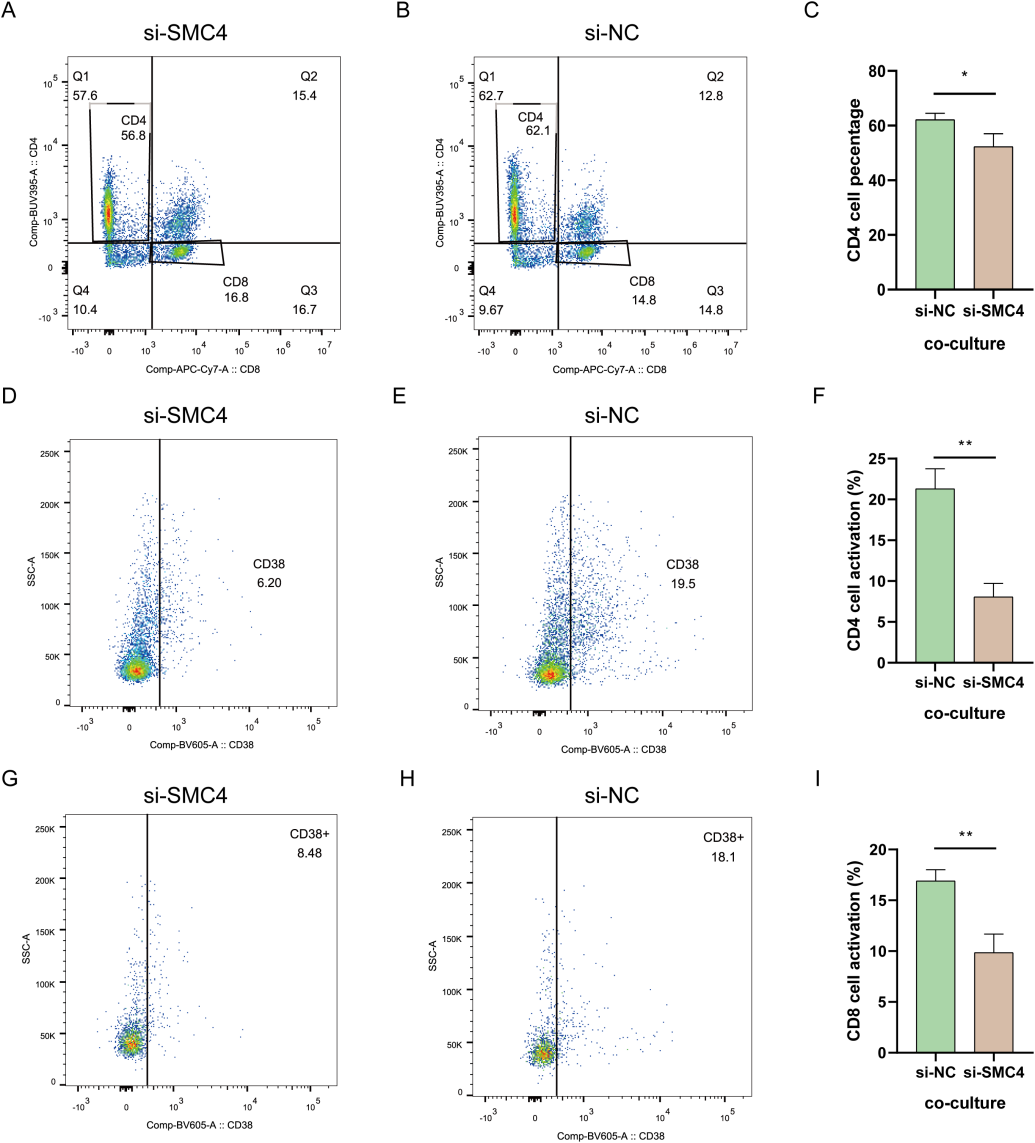
Statue of T cell activation. **(A–C)** T cell percentage. **(D–F)** CD4 T cell activation. **(G–I)** CD 8 T cell activation. **p < 0.01, *p < 0.05.

Our analysis showed a positive correlation between SMC4 expression in gliomas and CD4 T cell activation. To explore this further, we co-cultured SMC4 knockdown LN299 cells and control cells with T cells. The results indicated that LN299 cells with high SMC4 expression significantly increased the proportion of CD4 T cells (**Figures 13A-C**). However, SMC4 depletion led to a 13.3% reduction in CD4 T cell activation (**Figures 13D-F**) and a 9.62% decrease in CD8 T cell activation (**Figures 13G-I**). These findings suggest that SMC4 expression in glioma cells influences T cell cytotoxicity.

## 4 Discussion

GBM is a highly heterogeneous and aggressively vascularized malignancy, which contributes to its dismal prognosis (82). Within the GBM tumor microenvironment, immune evasion is a common phenomenon, with T-cell exhaustion playing a pivotal role. This exhaustion, often induced by persistent antigen exposure and chronic inflammatory states, results in diminished T cell functionality and immune escape (83). Exhausted T cells exhibit upregulation of inhibitory receptors such as PD-1, and their binding to PD-L1 on tumor cells dampens T cell responses, a mechanism central to immune checkpoint regulation and tumor immune evasion (84, 85).

Our research indicates that targeting the disulfidptosis-T cell exhaustion (Tex) network can modulate PD-L1 expression in glioblastoma cells (**Figure 11C**). Disulfidptosis, a regulated form of cell death, can potentially affect the survival and turnover of tumor cells. These observations suggest combining disulfidptosis inhibition with anti-PD-L1 therapy may improve clinical outcomes in glioblastoma treatment (**Figure 11A**). Additionally, disulfidptosis might promote immune evasion by influencing tumor cell metabolic pathways and stress responses. For instance, it could bolster antioxidant defenses in tumor cells, protecting them from immune-mediated damage driven by reactive oxygen species (ROS) (86). Moreover, disulfidptosis may alter the surface marker profile of tumor cells, thereby affecting immune recognition and clearance (87). Our findings also implicate the disulfidptosis-Tex axis in the T cell receptor signaling pathway (**Figure 7A**), which may contribute to creating an immunosuppressive environment, thereby facilitating immune escape. The altered oxidative and metabolic states of tumor cells (**Figure 3E**) may further impair T-cell activity, promoting tumor progression and immune resistance.

Interestingly, our data show dynamic shifts in the disulfidptosis-Tex network during the malignant transformation of glioblastoma (**Figure 3C**). Single-cell RNA sequencing (scRNA-seq) revealed considerable cellular heterogeneity, with endothelial cells exhibiting the highest levels of disulfidptosis-Tex activity, underscoring their critical role in tumor progression (88, 89). Endothelial cells are essential for vascular processes such as angiogenesis and permeability (90), and aberrant angiogenesis is a key driver of tumor growth, invasion, and recurrence (91–93). In addition, the disulfidptosis-Tex network not only enhances the invasiveness of glioblastoma cells but also increases their sensitivity to chemotherapy (**Figure 10C**). Although our study did not include *in vivo* validation, cell communication analyses suggest that endothelial cells (ECs) significantly influence the disulfidptosis-Tex interaction. Elevated vascular permeability can facilitate glioblastoma metastasis (48), and our findings emphasize the intricate interactions within the glioblastoma tumor microenvironment. Identifying key signaling pathways and ligand-receptor pairs, such as PSAP-GPR37 and SPP1-(ITGA5+ITGB1), highlights the importance of intercellular communication in modulating tumor behavior. The disulfidptosis-Tex-endothelial cell network plays a central role in glioblastoma progression, indicating that targeting this axis may offer new therapeutic avenues by modifying the tumor microenvironment.

Establishing an 11-gene disulfidptosis-Tex signature across independent cohorts reinforces its potential clinical utility. However, tumor resistance mechanisms often undermine therapeutic approaches’ effectiveness (94, 95). The analysis of the disulfidptosis-Tex risk model in glioblastoma may help identify patient subgroups that are more likely to respond to treatment. Strong correlations between immune cell subsets and prognostic genes suggest that immunotherapy could provide a promising alternative for patients with tumor (96–98), especially those with poor responses to conventional chemotherapy or targeted therapies. Nevertheless, further exploration of the immune microenvironment in GBM is essential.

Disulfidptosis-Tex plays a pivotal role in modulating tumor cell metabolism by influencing enzymatic activities, inducing gene mutations associated with metabolic processes, and activating critical signaling pathways. Genes involved in apoptosis regulation, RNA dynamics (99–102), and cell cycle control, such as SMC4 (103, 104), are integral to glioblastoma cell survival and proliferation, with our data suggesting that SMC4 significantly contributes to tumor cell growth and migration by regulating the cytoskeleton and maintaining mitochondrial integrity, as evidenced by increased ROS and altered mitochondrial membrane potential upon its inhibition. Additionally, genes like COL4A1, PLOD2, and PXDN (105–108), which participate in extracellular matrix remodeling, alongside immune-related genes such as CYFIP2, EMP3, and HLA-B (109–114), are instrumental in modulating the tumor microenvironment and facilitating immune evasion. These genetic interactions underscore the complexity of disulfidptosis-Tex’s role in glioblastoma progression. Our 11-gene disulfidptosis-Tex model holds promise as both a prognostic biomarker and a potential therapeutic target. By modulating T cell exhaustion, these genes may significantly influence the responsiveness of glioblastoma to immunotherapies. For instance, the observed upregulation of PD-L1 following the inhibition of disulfidptosis-Tex genes like SMC4 suggests a potential feedback mechanism that could be exploited to enhance the efficacy of PD-1/PD-L1 inhibitors. Furthermore, altered immune cell infiltration, such as decreased activation of CD4 and CD8 T cells upon SMC4 inhibition, highlights the intricate balance between tumor cell death pathways and immune surveillance. One hypothetical pathway is that SMC4 interacts with signaling molecules involved in the PD-1/PD-L1 axis, thereby affecting immune checkpoint regulation and T cell exhaustion. Additionally, SMC4 may influence the expression of chemokines or cytokines that attract or activate T cells within the tumor microenvironment. Understanding these interactions provides a foundation for developing combination therapies that simultaneously target disulfidptosis pathways and bolster immune responses, thereby improving therapeutic outcomes for glioblastoma patients. Future studies should delve deeper into the roles of these genes in disulfidptosis-Tex and glioblastoma progression, investigating the precise molecular mechanisms by which SMC4 operates and its interplay with immune cells, to fully elucidate their mechanisms and therapeutic potential.

While our study provides valuable insights into the role of disulfidptosis-Tex in glioblastoma progression and immune cell impairment, it is important to acknowledge several limitations that warrant further investigation. Primarily, our research relies heavily on multi-omics data, and the cellular communication mechanisms identified may not fully replicate the complexities of the actual glioblastoma immune microenvironment. The relatively small sample size within our glioblastoma cohort also limits the robustness and generalizability of our prognostic models, underscoring the necessity for validation in larger, independent cohorts. Additionally, our cellular experiments were exclusively conducted on glioblastoma cell lines, which restricts our ability to explore the effects of disulfidptosis on exhausted T cells directly. This narrow focus highlights the need for future studies to prioritize the biological impact of disulfidptosis on T-cell exhaustion and to incorporate diverse experimental models, including multiple glioblastoma cell lines and *in vivo* systems, to better mimic the tumor microenvironment. Furthermore, our 11-gene disulfidptosis-Tex prognostic model, while demonstrating strong predictive power within the training and internal validation cohorts, has yet to be validated externally due to the unavailability of independent datasets with comprehensive transcriptomic and clinical information.

Future research should aim to validate this prognostic signature in independent and diverse patient populations to confirm its clinical utility and generalizability. Prospective studies are also essential to assess the model’s effectiveness across various clinical settings, thereby enhancing its applicability and reliability. Given the inherent complexity of glioblastoma and the intricate interactions between disulfidptosis and other biological processes, extensive experimental validation is crucial to substantiate our findings and to fully elucidate the mechanistic pathways involved. Addressing these limitations in future investigations will not only strengthen the validity of our current findings but also pave the way for the development of more effective and personalized therapeutic strategies for glioblastoma patients.

## 5 Conclusion

Disulfidptosis-Tex genes are pivotal in regulating glioblastoma progression and immune cell infiltration, offering a novel strategy to modulate T cell exhaustion and enhance the efficacy of anti-glioblastoma therapies. Our research advances the understanding of how the disulfidptosis-Tex network contributes to glioblastoma progression and highlights potential therapeutic approaches targeting this pathway. These findings open up new possibilities for targeted interventions aimed at improving treatment outcomes for glioblastoma patients.

## Data Availability

The original contributions presented in the study are included in the article/supplementary material. Further inquiries can be directed to the corresponding author.
